# Cancer Stem Cells in Pancreatic Ductal Adenocarcinoma

**DOI:** 10.3390/ijms24087030

**Published:** 2023-04-10

**Authors:** Roman Bubin, Romans Uljanovs, Ilze Strumfa

**Affiliations:** 1 Faculty of Medicine, Riga Stradins University, 16 Dzirciema Street, LV-1007 Riga, Latvia; 034187@rsu.edu.lv; 2 Department of Pathology, Riga Stradins University, 16 Dzirciema Street, LV-1007 Riga, Latvia; romans.uljanovs@rsu.lv

**Keywords:** pancreatic ductal adenocarcinoma, cancer stem cells, epigenetics, signalling pathways, epithelial–mesenchymal transition

## Abstract

The first discovery of cancer stem cells (CSCs) in leukaemia triggered active research on stemness in neoplastic tissues. CSCs represent a subpopulation of malignant cells, defined by unique properties: a dedifferentiated state, self-renewal, pluripotency, an inherent resistance to chemo- and radiotherapy, the presence of certain epigenetic alterations, as well as a higher tumorigenicity in comparison with the general population of cancer cells. A combination of these features highlights CSCs as a high-priority target during cancer treatment. The presence of CSCs has been confirmed in multiple malignancies, including pancreatic ductal adenocarcinoma, an entity that is well known for its dismal prognosis. As the aggressive course of pancreatic carcinoma is partly attributable to treatment resistance, CSCs could contribute to adverse outcomes. The aim of this review is to summarize the current information regarding the markers and molecular features of CSCs in pancreatic ductal adenocarcinoma and the therapeutic options to remove them.

## 1. Introduction

Pancreatic ductal adenocarcinoma (PDAC) is a highly aggressive malignant tumour with a dismal prognosis. The current treatment options are insufficient because of the frequent occurrence of advanced disease [1,2], limited efficacy of surgical resection, as well as resistance to cytotoxic chemotherapy [3,4,5], radiation [6] and immunotherapy [7], resulting in an overall 5-year survival rate of approximately 7.1% [8]. According to Sung et al., the age-standardised incidence of pancreatic cancer is 5.7 cases per 100,000 in males and 4.1 cases per 100,000 in females, ranking it as the 13th most common cancer in the world. With the number of new deaths (466,003 in 2020) almost rivalling that of new cases (495,773 in 2020), it is the seventh leading cause of death among malignancies for both male and female patients [9]. Although the incidence of pancreatic cancer in some countries is considered stable [9], in other populations it is growing by about 1% per year, e.g., by 1.1% yearly in USA males and 1.0% in females within the time interval 2014–2018 [10]. High mortality, increasing incidence and resistance to therapy are the factors that make pancreatic cancer an unmet clinical need and, thus, an important target of scientific studies. In turn, PDAC is known to be the most common type of pancreatic cancer, constituting 85–90% of cases [1,3].

Tumour recurrence and treatment resistance are features classically associated with cancer stem cells (CSCs), a minor population of quiescent, self-renewing and pluripotent malignant cells [11]. CSCs are supposed to have a low proliferation rate, but, when dividing, they are able to restore the population of stem cells and to give rise to malignant daughter cells that constitute the main tumour mass [12]. Thus, when implanted in an experimental animal, stem cells can recapitulate the whole heterogeneity of the initial tumour. This feature of stem cells is reflected in the synonymous designation “tumour-initiating cells” (TICs). However, the ability to re-establish the tumour diversity in an experimental animal model should not be equated with the capacity to induce the malignant tumour in the patient. Indeed, four possible sources of CSCs are proposed, including normal stem cells, mature cells, cancer cells and fusion cells [13,14]. Currently, cancer stem cells are considered a state rather than an entity [13,15], likely being in dynamic balance with the general malignant population.

In 2007, Li et al. provided the first classic proof of the existence of a certain cell population possessing stem cell properties in pancreatic ductal adenocarcinoma. In this study, human pancreatic carcinoma cells were implanted into immunocompromised mice, allowed to grow and then sorted by flow cytometry, using specific markers. Scientists were able to identify a minor fraction (0.2–0.8%) of carcinoma cells, co-expressing CD24, CD44 and epithelial-specific antigen (ESA) and featuring high tumorigenicity, self-renewal capabilities and the ability to produce phenotypically diverse cancer cells [16]. Half a year later, a second report on cancer stem cells in pancreatic adenocarcinoma was published by Hermann et al., in which CSCs were defined by CD133 expression. Although CSCs were located in close proximity to epithelial cells, this subgroup showed no signs of cytokeratin expression. The tumorigenicity of isolated CD133+ and CD133− PDAC cells was studied on nude mice. Only 500 CSCs were required to produce a visible tumour in 3 weeks, while even a million CD133− cells were insufficient to initiate tumour growth during the same time frame. After treating PDAC cells with gemcitabine for 5 days, about 50% of surviving cancer cells expressed CD133, indicating towards higher treatment resistance in the CD133-positive population. The role of CSCs in PDAC metastasis was also studied, identifying chemokine (C-X-C motif) receptor 4 (CXCR4) co-expression as a driving factor of cancer cell migration and metastasis [17].

Conventional treatment such as chemo- and radiotherapy has a weak effect on CSCs because of the low proliferative activity s. quiescence; the increased expression of drug transporter molecules, which allows the elimination of toxic xenobiotics; the ability to scavenge reactive oxygen species; and efficient DNA repair mechanisms [12,18]. Cancer stem cells also successfully escape immune surveillance via the upregulation of programmed cell death ligand-1 (PD-L1) that is a negative regulator of T cells and, thus, suppresses activation of T lymphocytes; the downregulation of the natural killer (NK) activator molecule natural killer group 2D (NKG2D) ligand via tumour hypoxia, resulting in the loss of NK lymphocyte-mediated reactions against CSCs; and defects in the major histocompatibility complex MHC I, which affects the antigen presentation to T cells [12]. 

Because of the marked resistance to chemotherapy [19] and radiation [20], a specific therapeutic approach is required to target cancer stem cells. Currently, there are no guidelines for eliminating CSCs from PDAC. However, multiple studies have been carried out to determine potential drugs that could suppress stemness in pancreatic ductal adenocarcinoma.

The aim of the current review is to summarize the state-of-the-art information regarding the markers and molecular features of CSCs in PDAC and the therapeutic options to remove them.

## 2. Markers of Stem Cells in Pancreatic Ductal Adenocarcinoma

CSCs can be identified and/or isolated by using specific markers. The first known markers used for the identification of CSCs in PDAC were CD24, CD44 and ESA. It was confirmed by Li et al. [16] that CD24, CD44 and ESA-positive cells feature increased tumorigenicity and self-renewal capabilities. However, multiple new potential markers for identifying CSCs in PDAC emerged in the following years. 

### 2.1. CD44

CD44 is a non-kinase transmembrane glycoprotein that normally participates in cell–cell interactions, the adhesion of the cytoskeleton to the extracellular matrix and cell migration [21]. In addition, being expressed on normal embryonic stem cells, CD44 regulates embryogenesis. CD44 binds to several ligands, including collagen, chondroitin, osteopontin and fibronectin [22], but the main ligand of CD44 is hyaluronic acid—an abundant component of the extracellular matrix. The interaction between CD44 and hyaluronic acid induces conformational changes of the transmembrane molecule, followed by activation of multiple intracellular molecular signalling pathways (Ras, MAPK, PI3K) that ultimately upregulate cellular proliferation, adhesion, migration and, in case of malignant cells, invasion.

In different cancers, the classic features of tumour-inducing cells have been shown in animal experiments using CD44-positive malignant cells. As few as 100 CD44+ neoplastic cells are sufficient to initiate tumour in nude mice [21]. Regarding the experimental animal studies of PDAC, CD44 was among the stemness markers successfully applied in the classic study by Li et al. [16] to identify the fraction of carcinoma cells, featuring high tumorigenicity in a mouse model along with self-renewal capabilities and the ability to produce phenotypically diverse cancer cells.

In pancreatic ductal adenocarcinoma, CD44 is upregulated in comparison to normal tissues [23]. A high expression of CD44 has been reported in 37–42.7% of PDAC cases by Hou et al., 2014, and more recently by Askan et al., 2021 [24,25]. In both studies, cases were classified as CD44-positive if the expression exceeded the average level. Askan et al. defined the median value by a score, based both on staining intensity (four levels: none versus weak versus moderate versus strong) and expression range (none versus 1–10% versus 11–50% versus 51–80% versus 81–100%) while Hou used the mean count of positive cells and found it to be 9.71%. Thus, in both studies, the numbers of CD44-expressing cells significantly exceeded the burden of TICs, previously identified to be at the level of 0.2–0.8% for carcinoma cells [16,24,25]. Hence, the mere presence of CD44 is insufficient to detect cancer stem cells. Double reactivity with another cancer stem cell marker, e.g., CD24 or CD133, would be necessary. Indeed, CD133 has been explored as a CSC marker in pancreatic adenocarcinoma since the very first studies [17]. 

Considering the features of CSCs, the prognostic and/or predictive value of stem cell markers is also of interest, although the association is likely to be indirect and expression levels in human tumour tissues are influenced by a rich network of other factors [21]. The intense co-expression of CD44 and CD133 correlates with adverse disease-free survival. High levels of CD44/CD133-positive cells are associated with CD204-expressing M2-type tumour-associated macrophages that generally promote tumour development, and a high co-expression of all three markers is associated with a worse overall and disease-free survival [24]. In addition to the negative prognostic value, CD44 is associated with treatment resistance/failure. Thus, after palliative chemotherapy, circulating PDAC cells are enriched with CD44 [26]. Similarly, gemcitabine increased CD44 expression in PDAC implants in nude mice [22].

In summary, CD44 shows tumour-inducing features, upregulation in PDAC compared to non-neoplastic pancreatic parenchyma, as well as negative prognostic impact and features consistent with drug resistance. CD44 levels can define stroma characteristics and the spectrum of tumour-infiltrating immune cells.

### 2.2. Epithelial Cell Adhesion Molecule (Epithelial Specific Antigen)

Epithelial cell adhesion molecule (EpCam)**,** alternatively named epithelial specific antigen (ESA), has been found in various epithelial tumours as well as in acute myeloid leukemia. Its function in cancers is associated with the ability to activate transcription of genes that are responsible for cell proliferation (c-Myc and cyclins A and E), and to down-regulate immune response by reacting with leukocyte-associated immunoglobulin-like receptor 1 LAIR1 [27,28].

Wang et al. evaluated the expression of epithelial cell adhesion molecule in nasopharyngeal carcinoma and inflamed nasopharyngeal tissues. Based on qRT-PCR, carcinoma featured higher levels of EpCam than non-tumorous nasopharyngeal tissues. High levels of EpCam were associated with an increased frequency of metastatic spread: 42.9%, contrasting with 8.3% of patients having low levels of EpCam [29]. 

Western blotting was performed to evaluate whether the link between EpCam and the epithelial–mesenchymal transition (EMT) exists. When compared to the control group, EpCam-expressing cells had lower levels of epithelial markers, including E-cadherin, but mesenchymal markers N-cadherin, vimentin and β-catenin were upregulated. In addition, assessing stem cell markers via Western blotting revealed increased levels of ABCG2, CD44, Nanog, Oct4 and SNAIL2 [29]. Thus, EpCam expression parallels EMT, cancer metastasis and presence of other stemness markers. 

### 2.3. CD133

CD133 is a transmembrane glycoprotein that is involved in cell membrane organisation. It has been demonstrated in different cancers showing a close association with the Wnt/β-catenin pathway [21]. In a mouse model, sorted PDAC cells featuring high levels of CD133 were able to induce the tumorigenesis at low cell numbers (500 cells), while even 10^6^ patient-derived cells failed to initiate the tumour growth [17]. Thus, CD133-positive cells function like tumour-initiating cells. In addition, the low fraction of CD133 in normal pancreatic tissues and PDAC is consistent with the expected stem cell burden [17]. CD133-positive PDAC cells show increased survival when subjected to chemotherapeutic treatment [30]. 

However, some features of CD133-positive cells contrast with the CSC concept, e.g., CD133-overexpressing tumour cells showed higher levels of telomerase and higher proliferative activity [30]; lower CD133 expression was observed after neoadjuvant chemoradiotherapy of pancreatic cancer [31]. Nevertheless, CD133 has been successfully used as a CSC marker in pancreatic ductal adenocarcinoma [32].

Via reacting with histone deacetylase HDAC6, β-catenin and α-tubulin, CD133 stabilizes β-catenin that leads to the activation of the Wnt signalling pathway, augmenting the epithelial–mesenchymal transition (EMT). In turn, EMT promotes tumour invasiveness [33]. Clinically, CD133 expression in human PDAC tissues correlates with an increased rate of lymphatic invasion and lymph node metastasis, and a lower 5-year survival [34]. 

### 2.4. CXCR4

Chemokine (C-X-C motif) receptor 4 (CXCR4) has been evaluated in PDAC stem cells along with CD133 [17]. CXCR4 is normally detected in various cell types ranging from macrophages and lymphocytes to neurons and microglia. It functions as part of the CXCR4/CXCL12 axis, which is involved in both human embryonic development and the pathogenesis of inflammation [35]. In cancer, the CXCR4/CXCL12 axis is suggested to contribute to the viability of neoplastic cells, angiogenesis and metastatic activity of malignant cells. Within CSCs of pancreatic ductal adenocarcinoma, the combined expression of CD133 and CXCR4 correlated with an increased ability for invasion and metastatic spread that could be prevented by the blockage of CXCR4 [36].

### 2.5. CD24 

CD24 is a mucin-like glycosylphosphatidylinositol-anchored small membrane protein [37]. In PDAC research, CD24 was successfully implemented as one of the stem cell markers (along with CD44 and ESA) in the first animal studies of stemness in pancreatic ductal adenocarcinoma [16]. However, similarly to CD133, the expression of CD24 increases proliferation [38]. The role of CD24 in carcinogenesis is complex. Besides the proliferative effects, CD24 promotes binding to P-selectin and activates integrins A3B1 and A4B1, thus facilitating cancer cell adhesion to fibronectin, laminin and collagen I and IV. Via these mechanisms, CD24 supports motility, spreading, invasion and metastasis [38]. It is also linked to the regulation of the activity of the Wnt/β-catenin pathway [39].

### 2.6. ALDH1

Stem cells in cancer are characterised by the expression of aldehyde dehydrogenase 1 (ALDH1). Aldehyde dehydrogenases are intracellular enzymes that catalyse the oxidisation of aldehydes [36]. In particular, ALDH1A1 acts as a catalyst for the oxidation of retinol to retinoic acid. ALDH1 activity contributes to CSC survival by reducing the amount of reactive oxygen species (ROS), since ROS inhibit both DNA methylation and repair. Lower levels of reactive oxygen species ensure that cancer stem cells have a higher chance to avoid apoptosis [40]. In comparison with ALDH1-negative cells, ALDH1-positive cancer cells have a higher capacity to induce experimental tumours at low cell numbers [36]. ALDH has been successfully used as a CSC marker in experimental studies of pancreatic ductal adenocarcinoma [32]. However, the prognostic value of ALDH expression in PDAC is controversial [40].

### 2.7. C-Met

Single-pass tyrosine kinase c-Met is another marker of CSC in PDAC. c-Met is a receptor for its ligand hepatocyte growth factor HGF. Normally, c-Met is involved in embryogenesis and wound healing [41]. The high expression of c-Met in PDAC cells was associated with CSC behaviour: a higher capacity to form tumour spheres and to initiate tumours contrasting with an inability to develop spheroids by c-Met negative cells. As a stem cell marker, c-Met shows a higher accuracy in association with CD44 since the co-expression of both markers identified malignant cells with a higher tumour-initiating capacity [42].

### 2.8. DCLK1

Microtubule-associated doublecortin-like kinase 1 (DCLK1) was first described in brain tissues. Later, this protein was found in different carcinomas, including PDAC. In the pancreatic gland, DCLK1 is expressed in the acinar epithelium. DCLK1-positive cells ensure pancreatic regeneration, e.g., after injury or in chronic pancreatitis, but *K-Ras* mutations in these cells induce carcinoma. DCLK1-expressing PDAC cells show tumour-initiating features and form organoids [18].

In cancer stem cells, DCLK1 promotes angiogenesis in a hypoxic environment, since the expression of this protein is upregulated by low oxygen supply, while reducing DCLK1 negatively influences angiogenesis. There is also evidence of DCLK1 involvement in the regulation of epithelial–mesenchymal transition, as the inhibition of DCLK1 expression resulted in the downregulation of EMT transcription factors [18].

## 3. Cancer Stem Cells: Assays 

In the tumour sphere formation assay, the ability of cancer cell lines to form spherical colonies is evaluated when tumour cells are cultivated under low-attachment conditions [43]. The active formation of tumour spheres suggests self-renewal capabilities and is, thus, considered a hallmark of CSCs [43,44].

The side population assay evaluates cell capability of evicting Hoechst 33342 dye. Cells with an efflux of Hoechst are considered to possess stem-like capabilities, being able to eliminate toxic xenobiotics [44]. In pancreatic ductal adenocarcinoma, Broeck et al. confirmed the presence of a side population and assessed the expression of CSC markers within it. Isolated side population cells were overexpressing CD44, CD133 and CXCR4, as well as EpCam and CD24 [45].

To test the resistance to chemotherapy and/or hypoxic conditions, cancer cells are subjected to either chemotherapeutic agents, the lack of oxygen or both. CSCs are characterised by a higher resistance than the bulk of the tumour.

Density gradient configuration represents a method to evaluate the physical features of CSCs. Assessing stem cell markers in cells with a high nucleus-to-cytoplasmic ratio and a low number of organelles, more than 80% of cells were EpCam- and CD133-positive. The immature cells also featured the highest tumorigenic potential [46]. Similarly to tumour sphere evaluation, this approach does not require preliminary choice of stem cell markers that occasionally is controversial [46].

In fluorescence-activated cell sorting, marker-specific fluorescent antibodies are used to distinguish a certain cell population via the characteristic surface markers [46]. The magnetic activated cell sorting is also based on cell separation in accordance with the presence or absence of certain surface markers, but the magnetic field is used for sorting, and magnetic beads are applied to a cell culture. As a result, cells lacking markers of interest are filtered out [46,47].

## 4. Signalling Pathways in PDAC CSC

Stem cells in pancreatic adenocarcinoma are characterised by the aberrant activation of multiple signalling pathways, which are usually active during embryonic development. Via inducing abnormal signalling through Hedgehog, Wnt, Notch, JAK-STAT, Nodal/Activin and Hippo pathways, CSCs are able to maintain their unique ability of self-renewal, while simultaneously gaining resistance against chemo- and radiation treatment, increased tumour-inducing capacity and the ability to metastasize [48]. 

### 4.1. Notch Pathway 

**Figure 1:**
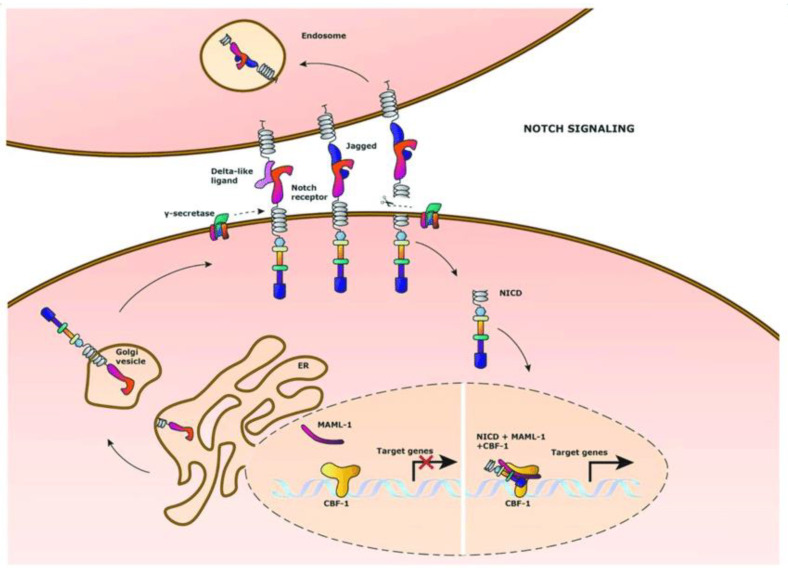
Notch signalling pathway. Figure replicated from [53] under Creative Commons license, provided at https://creativecommons.org/licenses/by/4.0/ (accessed on 29 January 2023). Changes have been made to the figure legend.

Notch signalling (Figure 1) pathway can act either as prooncogenic mechanism, or as a tumour suppressor [49,50,51]. During normal development, the Notch pathway is responsible for regulating embryogenesis, cell differentiation, programmed cell death and proliferation. In non-malignant adult human pancreatic tissues, the activity of this pathway is absent; pancreatic cancer cells, however, express Notch-associated markers (including Notch receptors, ligands and targets) and react to the inhibition of the aforementioned pathway [51]. 

The Notch pathway includes five Notch ligands: Jagged 1 and 2 (JAG1 and JAG2), as well as Delta-like 1, 3 and 4 (DLL1, DLL3 and DLL4); and four transmembrane Notch receptors: Notch 1–4 [52]. Activation of the classical Notch pathway is triggered by ligand binding to the receptor, usually as an interaction between two cells. The downstream events involve a chain of Notch receptor cleavage reactions, finally releasing an intracellular domain that translocates to the nucleus where it interacts with CBF1/suppressor of hairless/LAG1 complex CSL. Binding between the Notch intracellular domain NICD and CSL removes co-repressors from CSL, attracts co-activator complex and, thus, induces transcription of target genes, e.g., Nanog and others [48]. Notch signalling is considered non-canonical if Notch activity is initiated by other ligands, or triggered in the absence of ligand binding, or involves different intranuclear events [52].

In pancreatic ductal adenocarcinoma, CSCs express Notch 1 and Notch 2 [51]. Notch1 overexpressing PDAC cells had a higher potential to form tumour spheres with an elevated expression of CSC markers CD44 and EpCam [42]. The contribution of the Notch pathway to PDAC stem cell self-renewal capacity develops via the upregulation of several proteins, including Sox2, cMyc, Oct-4, survivin and Nanog [48].

### 4.2. Hedgehog Signalling Pathway 

During embryogenesis, the Hedgehog molecular pathway (HH) is involved in a normal development of the pancreas. In pancreatic cancer cells, HH is reactivated and promotes tumorigenesis [48]. One of the HH pathway ligands, Sonic hedgehog (SHH), was expressed by a majority (70%) of pancreatic cancers, while also being present in CSCs of pancreatic ductal adenocarcinoma [48].

**Figure 2:**
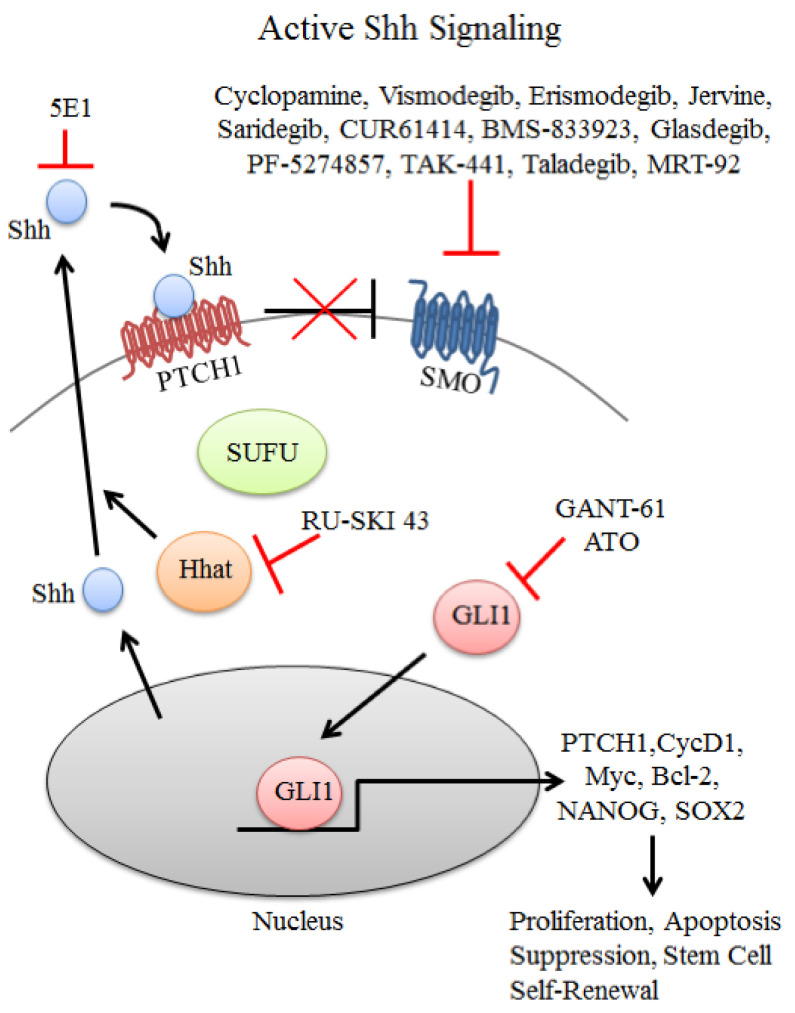
Hedgehog signalling pathway. Figure replicated from [55] under Creative Commons license, provided at https://creativecommons.org/licenses/by/4.0/ (accessed on 29 January 2023). Changes have been made to the figure legend and the figure has been modified.

The Hedgehog molecular pathway (Figure 2) is controlled by the transmembrane receptors Patched 1 (PTCH1) and Smoothened (SMO), which exhibit inhibitory and activating functions, respectively. In the absence of an activating ligand, PTCH1 keeps SMO inactive. The HH pathway becomes activated by the binding of ligand SHH to receptor PTCH1, releasing the previously bound SMO molecule. The downstream events result in the activation of the glioma-associated oncogene 1 (GLI 1), that enters the nucleolus and induces the transcription of genes responsible for cellular self-renewal, proliferation, and chemoresistance. The HH pathway can be blocked by arsenic trioxide (used for the treatment of acute promyelocytic leukaemia and solid tumours) as well as by vismodegib, representing a small molecule (2-arylpyridine class) inhibitor of SMO [11,54]. 

Vismodegib has been evaluated in a pancreatic cancer model. It was able to inhibit cellular viability and induce apoptosis both in pancreatic cancer cell lines and stem cells. Inhibition of the HH pathway also changed the expression of various proteins: the expression of pro-apoptotic FAS was increased, while markers associated with cell survival, i.e., Bcl-2 and platelet-derived growth factor alpha (PDGFRα), were downregulated [54].

### 4.3. Wnt Pathway 

**Figure 3:**
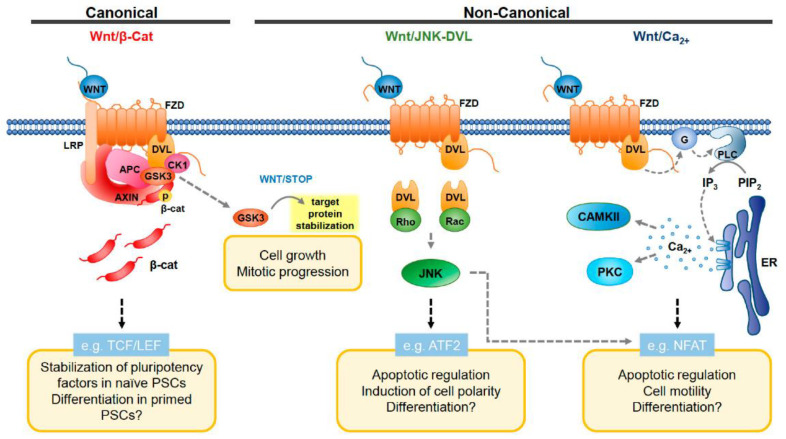
Canonical and non-canonical Wnt signalling. In canonical Wnt pathway, the binding of Wnt ligand and Frizzled (Fzd) receptor results in a loss of phosphorylation of β-catenin. β-catenin then migrates to the cell nucleus to trigger gene transcription. As for non-canonical JNK-DVL signalling, the reaction with the Frizzled receptor activates DVL, which then initiates the activity of Rho and Rac. As a result, JNK signalling is triggered. In calcium-dependent signalling, the reaction between the ligand and receptor is followed by the activation of the G-protein, then by elevation of intracellular Ca^2+^ levels [59]. Figure replicated from [59] under Creative Commons license, provided at https://creativecommons.org/licenses/by/4.0/ (accessed on 29 January 2023). Changes have been made to the figure legend.

Since the identification of the *wingless* gene in *Drosophila melanogaster* approximately 40 years ago, the Wnt molecular pathway (Figure 3) has become one of the most studied signalling chains. Its functions include the regulation of organ development and cell differentiation in embryogenesis [56], as well as cell proliferation, migration and carcinogenesis. Currently, canonical s. beta-catenin-dependent and non-canonical s. beta-catenin-independent Wnt signalling is distinguished [57,58]. The canonical Wnt pathway involves signalling via the Frizzled receptor (Fzd) and the low-density lipoprotein receptor-related protein 5/6 (LRP5/6) that finally influences the activity of nuclear β-catenin. The non-canonical Wnt pathway involves the Fzd receptor along with the tyrosine kinase-like orphan receptor (ROR)1, ROR2 and receptor tyrosine kinase (RYK) co-receptors, and the final target is the activation of Rho GTPases, including Rac1, RhoA and c-Jun N-terminal kinase (JNK) that control rearrangements in the cytoskeleton and gene expression. Another form of the β-catenin-independent non-canonical Wnt pathway is the Wnt-Ca^2+^-dependent signalling cascade [57]. 

**Figure 4:**
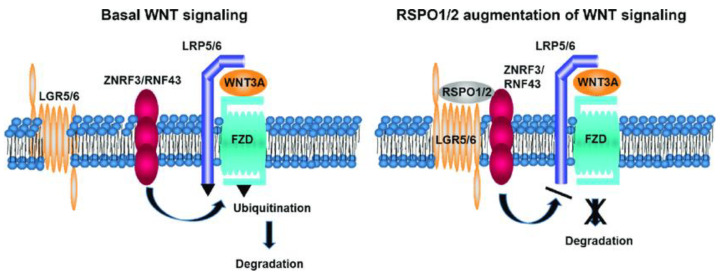
Wnt signalling, augmented with R-spondin. Figure replicated from [62] under Creative Commons license, provided at https://creativecommons.org/licenses/by/3.0/ (accessed on 29 January 2023). Changes have been made to the figure legend.

In pancreatic ductal adenocarcinoma, canonical Wnt signalling is regulated with the help of R-spondin proteins RSPO (Figure 4), which modulate LRP6 [60]. Phosphorylation of LRP6 protein leads to an induction of Wnt signalling, although the activity of the said cascade is, in turn, regulated by LRP6 internalisation, provided by DKK proteins. Through the influence of RSPO, the process of internalisation is downregulated, thus enhancing Wnt signalling in cancer cells. Highly tumorigenic and CSC-marker-overexpressing pancreatic cells exhibit high levels of RSPO2; cancer stem cells with a higher Wnt activity also possess increased tumorigenicity [61].

Elevated expression of non-canonical Wnt ligands Wnt2 or Wnt5A in pancreatic cancer cells promotes stemness, since non-canonical Wnt drives the epithelial–mesenchymal transition, as well as increases chemoresistance: Wnt5A signalling contributes to cancer cell survivability by reducing the chemotherapy-induced apoptosis [60].

### 4.4. Hippo Pathway 

**Figure 5:**
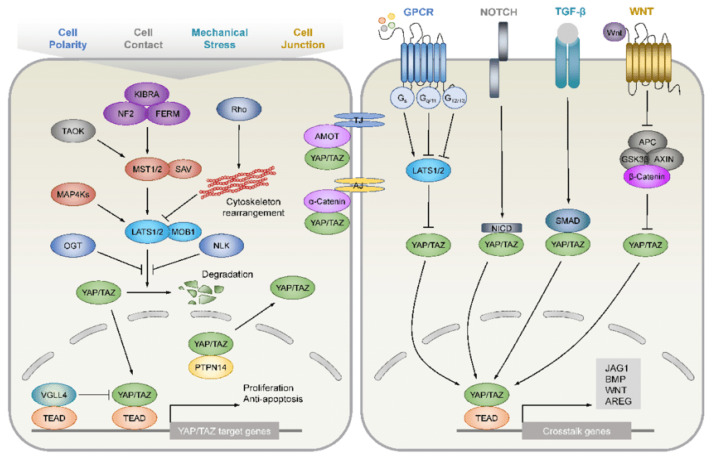
Hippo pathway and its crosstalk with other signalling pathways. Figure replicated from [65] under Creative Commons license, provided at https://creativecommons.org/licenses/by-nc/4.0/ (accessed on 29 January 2023). Changes have been made to the figure legend.

During the development of pancreatic tissues, the Hippo pathway regulates cell differentiation and proliferation, as well as apoptosis and the self-renewal of stem cells. The key components of Hippo signalling (Figure 5) are mammalian serine/threonine kinases MST1 and MST2, as well as large tumour suppressors LATS1 and LATS2 [63]. MSTs activate LATS kinases via phosphorylation, which, in turn, allows the latter to phosphorylate transcription coactivators YES-associated protein YAP and transcriptional co-activator with PDZ-binding motif TAZ. Phosphorylated forms of YAP and TAZ are unable to initiate transcription because access to the cell nucleus is blocked, and these proteins may accumulate only in the cytoplasm. In contrast, the absence of active LATS leads to a nuclear accumulation of YAP and TAZ, and their subsequent reaction with TEA transcriptional factors TEAD 1–4 allows for transcription to happen [63,64]. The Hippo pathway is subjected to multiple control mechanisms as well as crosstalk with other molecular pathways that can independently influence YAP and TAZ. 

In pancreatic ductal adenocarcinoma, overexpression of YAP has been identified, which might be initiated by the *K-Ras* mutation. However, *K-Ras* activation is not mandatory for the survival of PDAC cells; therefore, alternative mechanisms for YAP induction exist in pancreatic cancer [63]. In general, YAP and TAZ in tumours can be up-regulated by a wide scope of mechanisms, including amplification of the *YAP* gene, inactivation of the Hippo pathway, activating *K-Ras* mutations or interaction with other molecular pathways, including Notch, Wnt or TGFβ [66]. 

Upregulation of YAP and TAZ in different cancers (oesophageal squamous cell carcinoma, head and neck squamous cell carcinoma bladder cancer, lung cancer) is associated with increased expression of stemness markers CD44, CD133, SOX2, SOX9, Nanog, ALDH, higher chemoresistance, higher expression of multi drug resistance efflux transport ATP-binding cassette transporter protein ABCG2 and more active colony formation [66]. Similarly, in pancreatic cancer, nuclear translocation of YAP, induced by hepatocyte growth factor secreted by pancreatic stellate cells, increased the tumour sphere formation ability as well as the expression of stemness markers Nanog, OCT-4 and SOX-2 [67].

An additional underlying mechanism is the interaction between PDAC and its stromal component. PDAC cells secrete tissue transglutaminase 2: an enzyme capable of stimulating the proliferation of cancer-associated fibroblasts from activated stellate pancreatic cells. The increased stromal component of the cancer leads to the upregulation of YAP/TAZ [68,69]. Another link between the Hippo pathway and pancreatic CSCs is based on the ability of YAP and TAZ to mediate the epithelial–mesenchymal transition in cancer cells. Without the intranuclear accumulation of TAZ and YAP, epithelial tissues are prevented from gaining mesenchymal phenotype through TGF-β-induced EMT; a lack of EMT negatively impacts the stemness of carcinoma [70]. 

### 4.5. JAK/STAT Signalling

**Figure 6:**
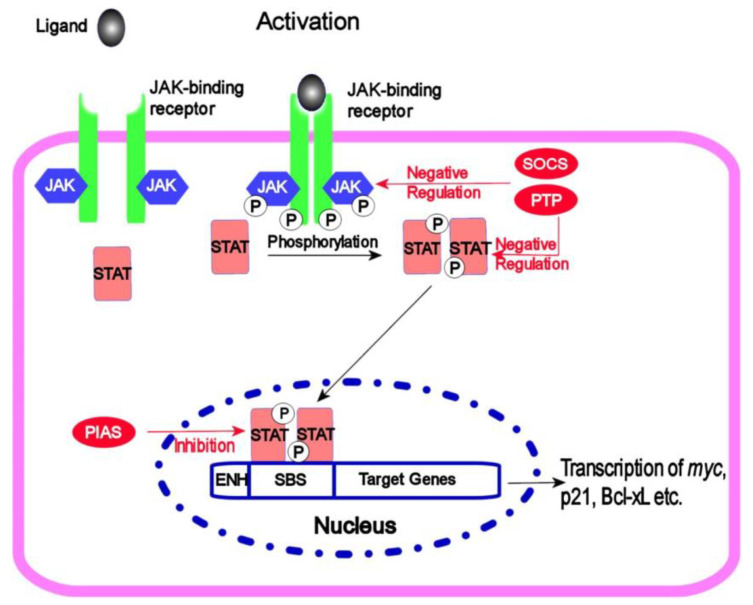
JAK-STAT signalling. Figure replicated from [72] under Creative Commons license, provided at https://creativecommons.org/licenses/by/2.0 (accessed on 3 February 2023). Changes have been made to the figure legend.

The Janus kinase/signal transducers and activators of the transcription (JAK/STAT) molecular pathway are triggered by cytokines. The regulatory effects influence cell proliferation, differentiation, apoptosis and immune regulation. The JAK/STAT pathway (Figure 6) consists of three main components: (1) Receptors that binds cytokines and growth factors. The receptors lack kinase activity themselves but are associated with tyrosine kinase. (2) Tyrosine kinase family JAK (including JAK1, JAK2, JAK3 and Tyk2). When a ligand engages the JAK/STAT pathway receptor, JAK activation leads to the phosphorylation of tyrosine residues of various target proteins. (3) The family of transcription factors STAT (at least seven members: STAT1, STAT2, STAT3, STAT4, STAT5a, STAT5b and STAT6). STAT is activated via phosphorylation of a single tyrosine residue, which triggers dimerisation. Subsequently, STAT dimer changes its localisation from the cytoplasm to the nucleus where it functions as a transcription factor [71]. 

For pancreatic ductal adenocarcinoma, it is rather common (30–100%) to express one of the STAT family proteins, namely, STAT3. Janus kinases activate this protein by phosphorylating Tyr705 [73]. The activation of STAT3 in pancreatic ductal adenocarcinoma is associated with autocrine and/or paracrine epidermal growth factor receptor signalling, since the selective inhibition of epidermal growth factor receptor results in a loss of activated STAT3 expression. Autocrine and/or paracrine interleukin-6 can also trigger STAT activity. Through the JAK-STAT pathway, PDAC cells gain an increased proliferation rate, as this signalling accelerates the cell cycle (G1 to S transition) [74]. In an animal model, the functional inactivation of STAT3 is associated with reduced tumour growth, if neoplastic cells are injected in nude mice [74]. Without the signalling activity of STAT3, pancreatic CSCs’ ability to form tumour spheres and maintain stemness is reduced, as was observed in the stemness model, designated to evaluate the role of p21-activated kinase 4 in the maintenance of stem cell features [75].

### 4.6. Nodal/Activin Pathway

**Figure 7:**
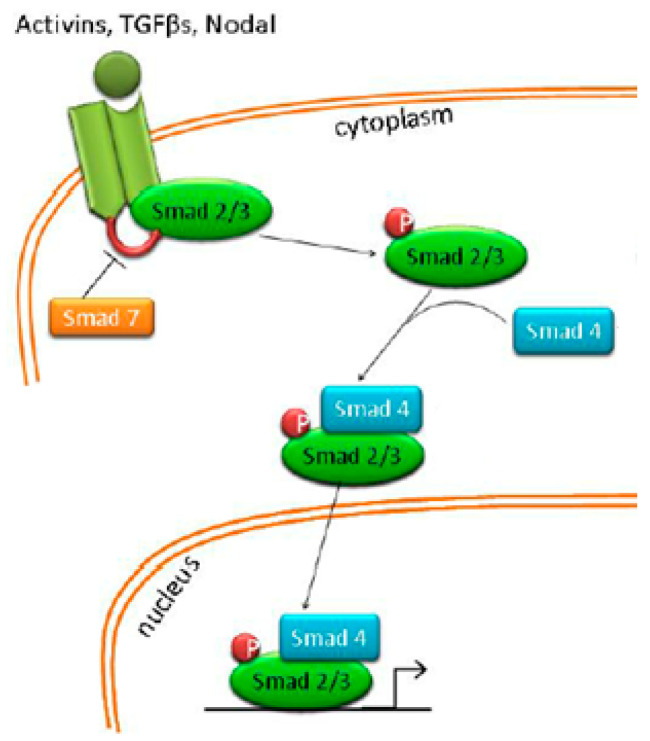
Nodal/activing signalling. Figure replicated from [78] under Creative Commons license, provided at https://creativecommons.org/licenses/by/3.0/ (accessed on 3 February 2023). Changes have been made to the figure legend and the figure has been modified.

The Nodal/Activin pathway (Figure 7) involves two types of Activin receptors. Nodal/Activin binding of type II Activin receptors (ActRII/IIB) allows the phosphorylation of type I receptors (ALKs 1–7). The activation of this pathway triggers the phosphorylation of Smad 2 and Smad 3 factors. Phosphorylated Smad factors merge into a complex, which then reacts with Smad 4, moves into the nucleus and triggers gene expression [76,77].

Stem cells of pancreatic ductal adenocarcinoma express markers associated with the activity of the Nodal/Activin pathway: CD133-positive cells overexpressed Cripto 1,3 Activin and Nodal proteins, as well as showed an increased expression of type I activin receptor ALK4 [79]. By studying Nodal/Activin signalling in pancreatic CSCs, Lonardo et al. discovered that downregulating this pathway through the inhibition of Alk4, Smad4 or Nodal resulted in a decreased sphere formation of pancreatic cancer stem cells, as well as reduced tumorigenicity [79].

### 4.7. K-Ras Mutation and Its Involvement in CSCs and Signalling Pathway Regulation 

*K-Ras* is a proto-oncogene, commonly activated in pancreatic ductal adenocarcinoma (~85% of PDACs had *K-Ras* mutations) and other malignancies. It belongs to a *Ras* oncogene family, along with *H-Ras* and *N-Ras*. *K-Ras* mutations are encountered significantly more frequently than *H-Ras* or *N-Ras*. Mutations in these genes results in a decreased hydrolysis of GTP, which prolongs the active state of the Ras-protein [80].

*K-Ras*-mutated cells have an increased capacity to form tumour spheres, when compared with *H-Ras* mutated cells, as well as a higher tumour-initiating capacity. Mutant *K-Ras* cells are characterised by a lower expression of Frizzled 8 (FZD8) receptor, involved in Wnt signalling [81].

To determine, whether Frizzled 8 expression had an impact on stem cell properties, knockdown of Fzd8 was performed in *H-Ras* cells, which resulted in an increased tumour sphere formation. After knockdown, *H-Ras* cells acquired increased tumorigenicity: on the 48^th^ day, 100% of nude mice developed a tumour after being injected with *H-Ras* cells versus 30% in the control group. Increased Frizzled 8 expression in *K-Ras* cells promoted calcium-dependent Wnt signalling, and reduced tumour sphere formation and tumour-initiating capabilities (in the *K-Ras* group with restored Frizzled 8 expression, 0 out of 10 recipients had developed tumour on the 36^th^ day, but in the control *K-Ras* group, tumours were found in 9 out of 10 recipients). *K-Ras* might also downregulate the activity of non-canonical Wnt signalling through the binding of calmodulin—one of the components of calcium-dependent signalling [81]. 

The involvement of *K-Ras* in cell stemness is not limited to Wnt signalling. Ji et al. identified the activation of the HH pathway via *K-Ras* mutations. The inhibition of *K-Ras* was performed to prove the link between activating the mutation of *K-Ras* and HH, and it resulted in decreased levels of Gli1, known as a transcriptional factor of HH pathway [82]. 

## 5. Metabolism of Stem Cells in Pancreatic Ductal Adenocarcinoma

The metabolism of cancer cells differs significantly from the biochemical events in non-malignant cells. There are two metabolic pathways for energy gain: oxidative phosphorylation and glycolysis. Normal cells usually use oxidative phosphorylation to produce ATP, and switch to glycolysis under anaerobic conditions. Cancer cells, however, are capable of aerobic glycolysis, modifying their metabolic pathway so that energy is generated via glycolysis, even in the presence of oxygen [83,84].

The metabolic phenotype of CSCs is a complex and controversial issue. Some authors have found that CSCs mainly use glycolysis as a means to generate ATP [85,86]; others have reported low glycolytic activity and a predominance of oxidative phosphorylation in CSCs [18,87,88]. Pancreatic cancer stem cells fall into the second category: while their non-stem relatives are mainly focused on glycolysis, downregulation of glycolysis yields no significant changes in PDAC CSCs. Further, the predominance of oxidative phosphorylation in CSCs was confirmed by a dramatic reaction to a knockdown of mitochondrial metabolism in CSCs, while depriving non-stem cells of oxidative phosphorylation resulted in negligible changes in ATP production. However, after metformin treatment, surviving stem cells not only acquired resistance to this agent, but also switched their metabolic phenotype to an “intermediate” state—tests revealed increased glycolytic activity, while partially preserving the mitochondrial metabolism. This metabolic switch is regulated by an MYC/PGC-1α balance. PGC-1α expression is triggered by the downregulation of MYC and drives the oxidative phosphorylation, while also inhibiting the glycolytic activity of tumour cells [89]. MYC, however, is a more complex regulator, capable of influencing both glycolytic and mitochondrial metabolic pathways [89,90]. 

The metabolism of PDAC CSCs has also been explored by Domenichini et al. They noted that upon stress, tumour spheres, representing a CSC-enriched population, maintained a low oxygen consumption rate, only mildly increasing the activity of glycolysis. In contrast, stressed non-stem cells increased both the activity of glycolysis and mitochondrial oxidative phosphorylation. However, the metabolic responses were heterogeneous, depending on the cell line [91].

## 6. Epithelial–Mesenchymal Transition and Its Association with Stem Cells in Pancreatic Ductal Adenocarcinoma

The epithelial–mesenchymal transition (EMT) is active during normal development and undergoes reactivation in malignancies. It is classified into three different functional types. Type 1 EMT is responsible for the production of primary mesenchyme, while type 2 is associated with regenerative processes, including fibrosis. Type 3 is active in malignancies, including PDAC, and is believed to be linked to cancer stem cells. This class of EMT promotes the migration of neoplastic cells, enhances the viability and invasiveness and allows for cancer cells to undergo an incomplete transition and, thus, acquire a complex phenotypical state between the epithelium and the mesenchyme [92].

In order to prove the EMT in cancer, a number of different markers were used. EMT is characterised by loss of epithelial morphology, functional features and markers (E-cadherin, integrins and cytokeratins) and the acquisition of mesenchymal features, including the expression of N-cadherin, vimentin and fibronectin. The transition is triggered by corresponding transcription factors SNAIL1, SNAIL2, ZEB1 and ZEB2, followed by a downregulation of E-cadherin. In the mesenchymal state, cancer cells are more resistant to gemcitabine, and unlike their epithelial counterparts, mesenchymal cells gain mobility, which allows for metastatic spread [93]. 

According to Zhou et al. and Scheel and Weinberg, the stromal component of carcinoma is responsible for the initiation of EMT [93,94]. TGF-β, which is secreted by pancreatic cancer cells, plays a major role in inducing the transition [95,96]. TGF-β triggers the EMT through the activation of the SMAD family. The activated complex, consisting of SMAD 2, 3 and 4, assumes an intranuclear position, triggering the expression of genes, which results in an elevated expression of SNAIL [56,97]. Subsequently, the EMT is initiated as a response to an increase in SNAIL transcription factors. SNAIL also downregulates the expression of epithelial E-cadherin and allows for the expression of ZEB1, which further drives the cells to undergo the transition [98,99].

There is a tendency towards a link between EMT and stem cells in cancer. Previously, it was noted that after undergoing EMT and acquiring a mesenchymal state, breast carcinoma cells actively expressed CSC marker CD44, and tumour sphere formation was also greatly enhanced [100]. In pancreatic cancer, it was shown that after the EMT, cancer cells expressed stemness markers, became capable of self-renewal and were able to initiate tumour growth at a much faster rate than their counterparts. The differences were clearly seen by comparing tumour initiation by pancreatic intraepithelial neoplasia (PanIN) cells: E-cadherin-positive PanIN cells were unable to form a tumour in 2 months, but cells of mesenchymal differentiation initiated tumour growth in four out of six animals. It took about 4 months for epithelial PanIN cells to achieve comparable results [101].

Multiple signalling pathways regulate the epithelial-to-mesenchymal transition. Song et al. studied the role of canonical Wnt and PI3K/Akt signalling in EMT. They reported that the inhibition of the respective pathways resulted in an upregulation of E-cadherin and a significantly reduced expression of N-cadherin and EMT transcription factors SNAIL1 and SNAIL2 in cancer cells [102].

The Hedgehog pathway is also involved in EMT regulation. Comparing pancreatic cancer spheres to the control group, the overexpression of Smoothened, Gli1, Shh, SNAIL and N-cadherin was found, but the E-cadherin level was lower. The knockdown of Smoothened was performed to study the effect of the HH pathway on tumour spheres. The assessment of EMT markers via RT-PCR revealed increased levels of E-cadherin; N-cadherin and SNAIL expression was reduced. After knockdown of Smoothened, the number of CD24 + CD44 + ESA+ cells decreased [103].

Regarding the Notch pathway, cells with active Notch 1 or Notch 4 expressed lower levels of E-cadherin. Compared with the Notch-negative control group, Notch+ breast cancer cells had higher levels of SNAIL2. The binding of CSL to SNAIL2 promoter regions further proved the association between the Notch molecular cascade and EMT. Jagged1-expressing cells also had a higher expression of SNAIL2 [104].

## 7. Epigenetic Events in Pancreatic Cancer Stem Cells

Epigenetic changes are alterations of gene expression without changes in DNA sequence. The epigenetic regulation of CSCs occurs via (1) DNA methylation, (2) alterations to the chromatin architecture that are either due to the post-translational modification of histones or the activity of polycomb group proteins, as well as (3) a modified spectrum of microRNAs [11,105].

The aberrant methylation of DNA is controlled by a family of DNA methyltransferases: DNMT1, DNMT2, DNMT3A, -3B and -3L [106]. Comparing the methylation differences between stem and non-stem cells of pancreatic carcinoma, CD133-expressing population of PDAC cells had higher methylation levels than CD133-negative cells. Elevated levels of DNMT1 expression were identified in CSCs via Western blotting. Comparing the expression of DNMT1 in CSCs and non-CSCs, the CD133-positive stem cell population had significantly higher levels of methyltransferase. The use of DNMT1 inhibitor zebularine significantly reduced the self-renewal capacity and expression of Oct3, SOX2, Nanog and KLF4. Another notable consequence of zebularine was its ability to promote differentiation among stem cells: after receiving zebularine, the CD133-negative population of cells had expanded, while numbers of CD133-positive cells were reduced. The hypothetic mechanism of the inhibition of DNMT1 is the downregulation of methyltransferase, causing an increased expression of microRNA-203 and -205, which are involved in differentiation. Additionally, zebularine can reactivate the expression of the miR-17-92 cluster, which is usually downregulated in cancer stem cells [62,107].

Alterations of the histone structure represent an additional mechanism of epigenetic regulation. Gemcitabine-resistant PDAC cells express higher levels of histone-modifying proteins. Among these enzymes, G9a was evaluated for its association with stemness. In PDAC cell cultures, the expression of G9a correlated with an upregulation of stem cell markers CD133, Lgr5 and nestin. To prove the stemness of chemoresistant G9a-expressing cells, a comparison was made between the bulk of PDAC cells and gemcitabine-resistant cells by the tumour sphere-forming capacity. As expected, self-renewal properties were significantly more prominent among the chemoresistant population. Researchers concluded that G9a might promote the resistance of cancer cells by elevating the expression of IL-8. After inhibiting G9a, the tumorigenic properties of chemoresistant cancer cell population were reduced [108]. The findings were verified by another group of scientists. Although the stemness was assessed by a different set of markers, reduced levels of G9a were accompanied by a loss of tumour-initiating cells [109].

The non-coding RNAs also participate in the regulation of stemness in PDAC. There are two major subgroups of non-coding RNAs: long non-coding RNAs (>200 nucleotides) and miRNAs. Among the long, non-coding RNAs (lncRNAs), HOTTIP and NORAD have been associated with CSCs in PDAC [106,110,111]. A subpopulation of pancreatic carcinoma cells, possessing self-renewal capabilities and expressing stem cell markers, was shown to overexpress HOTTIP. The link between this lncRNA and stem cells in PDAC is indirect and lies in its ability to elevate the expression of HOXA9, which drives the stem-like phenotype by activating the Wnt pathway. A knockdown of either HOXA9 or HOTTIP resulted in a significant loss of tumour-initiating potential. The tumour volumes of the said groups were much lower: by the 20th day, the CSC-induced tumours reached a volume close to 600 mm³, while the volumes of tumours induced by cells with blocked HOXA and HOTTIP were almost zero [110]. NORAD induces stemness in PDAC via its ability to suppress miR-202-5p-miRNA, which is capable of downregulating the expression of ANP32E and, thus, reducing the stemness markers and sphere-forming capabilities, along with the tumorigenicity of pancreatic CSCs [111].

miRNAs may exhibit different effects: some (such as the abovementioned miR-202-5p) act as tumour suppressors, while others contribute to tumorigenicity and invasiveness [112]. miR-21 is an oncogenic microRNA that is dysregulated in pancreatic cancer. The exact mechanism behind its function in CSCs is unclear; however, the silencing of miR-21 resulted in a downregulation of CSCs and mesenchymal EMT markers, but an elevated expression of E-cadherin [112,113].

MiR-34a, on the other hand, acts as a tumour suppressor. Its expression in pancreatic cancer cells and CSCs is reduced. After restoring the expression of this silenced miRNA in PDAC CSCs by chromatin-modifying agents, miR-34a activity promoted apoptosis and lowered cell proliferation in the stem cell population. The overexpression of mi-R34a was followed by the downregulation of VEGF, along with the proteins involved in the regulation of the cell cycle: cyclin D1 and CDK2. Another notable feature of the re-expression of mi-R34a is the inhibition of the Notch pathway. In comparison with the control group, cells with restored miR34a activity had a significantly lower expression of Notch genes Notch 1, Notch 3, JAG1 and HES1 [114].

## 8. PDAC Cancer Stem Cells: Treatment Options 

The mechanisms behind the viability of CSCs are diverse and most likely incompletely understood; therefore, cancer stem cells still represent a topic of active research. Currently, there is no possibility to overcome all the defensive mechanisms of CSCs with a single drug. Even more importantly, the spontaneous development of cancer stem cells from non-stem malignant cells precludes the possibility of isolated eradication of CSCs. However, multiple medications (Table 1) have been tested in order to determine their influence on pancreatic cancer stem cells. While not being confirmed for the treatment of PDAC patients, these drugs may serve as a starting point for discovering an effective and safe method of eliminating stem cell components of carcinoma within a complex approach.

### 8.1. Salinomycin 

Several antibiotics have shown efficacy in suppressing cancer stem cells. Gupta et al. identified that treating stem-like breast cancer cells with salinomycin decreased the overall CSC population, tumour sphere formation, as well as the expression of CSC-associated genes [115]. Its effectiveness in pancreatic cancer was proven by Zhang et al. who showed that salinomycin had a cytotoxic effect on PDAC cells and was able to disrupt sphere formation. The primary target of salinomycin in cancer is the CD133-expressing subpopulation of cancer cells, while the effects of salinomycin on CD133-negative tumour cells were not as notable [116]. This relatively selective anti stem-cell effect of salinomycin might be attributed to its ability to interfere with CSC signalling pathways and inhibit them: by preventing the phosphorylation of LARP6, salinomycin blocks the Wnt pathway [117].

### 8.2. Gramicidin A 

Gramicidin A is another antibiotic that is potentially suitable for treating CSCs of pancreatic ductal adenocarcinoma. It can reduce the self-renewal capacity of cancer stem cells, suppress proliferation, activate apoptosis and downregulate expression of CSC markers CD133, CD44, as well as CD47. These effects might be due to the gramicidin-mediated influence on cancer cell mitochondria, since changes in the mitochondrial ultrastructure and cell membrane were observed. However, gramicidin A is associated with toxicity, namely, haemolysis [118].

### 8.3. Chloroquine 

Anti-malarial medication chloroquine has shown positive results as an anti-CSC drug for pancreatic cancer. After receiving chloroquine, the ability of cancer stem cells to form spheres was reduced significantly, as was their number and tumorigenicity. Chloroquine downregulates CXCR4 expression and suppresses the activity of the Hedgehog signalling pathway via the reduction of Smoothened [119]—one of the key components of HH, which serves as a transcription initiator [22]. Moreover, chloroquine is also capable of inhibiting stromal signalling in PDAC, thus reducing the number of cancer-associated fibroblasts [119] that subsequently decrease tumour cell proliferation [68,69].

### 8.4. Aspirin

Aspirin is effective in reducing the resistance of PDAC to gemcitabine via the downregulation of Oct4, Nanog, SOX2 and ALDH1, which suppresses CSC self-renewal capacity. Another notable feature of aspirin is its inhibiting effect on NF–kb [120]. In cancer cells, NF–kb promotes the proliferation and resistance against apoptosis and, hence, the suppression of its activity increases the death rate of neoplastic cells [121]. 

### 8.5. Disulfiram

Another non-conventional drug that could potentially be applied for the treatment of PDAC CSCs is disulfiram, traditionally used to treat alcoholism. While not being effective against CSCs on its own, combining disulfiram with Cu results in a significant downregulation of ALDH+ cells of PDAC. By assessing the number of cells that expressed stem cell markers CD133, CD44, CD24 and ESA before and after therapy, it was found that the combination of disulfiram/Cu, chemotherapy and radiation was more potent against PDAC stem cells than chemotherapy accompanied by radiation. This anti-stem effect might be attributed to the ability of the disulfiram/Cu complex to inhibit NF-kb; however, when comparing disulfiram with dedicated inhibitors of NF-kb, disulfiram/Cu showed better results in suppressing the tumour sphere formation of CSCs [122].

### 8.6. EpCam/CD3 Bispecific T-Cell Engaging Antibody 

Cioffi et al. studied the effect of the EpCam/CD3 BiTE (Bispecific T-cell Engaging) antibody MT110 on pancreatic stem cells. They reported that after treatment with MT110, cancer cells were less inclined to form tumour spheres, indicating the loss of CSC capabilities. CSCs that were exposed to MT110 had a significantly reduced tumour-initiating capacity. To evaluate this, after 7 days of exposure to antibodies, the cells were implanted into immunodeficient mice and allowed to grow for 60 days. In comparison to the control group, the tumour volume of cells that received MT110 treatment was either negligible or absent in two out of three mice in an MT110 group, despite sphere formation not being completely disrupted. However, MT110 is not as effective at eliminating metastatic cells as it is against primary CSCs [123].

### 8.7. Metformin

Metformin, primarily used as a therapy in type II diabetes to reduce the resistance to insulin, might also be effective against CSCs in pancreatic cancer. In 2011, Bao et al. tested the effects of metformin on CSCs. During the experiment, chemoresistant cells were exposed to various doses of metformin, ranging from 5 to 30 mmol/L, and compared to the control group. While a dose of 5 mmol/L had negligible effect on the survival of gemcitabine-resistant cancer cells, treatment using 20 and 30 mmol/L of metformin significantly reduced the number of tumour cells. Exposure to large doses of metformin was highly disruptive to the process of self-renewal and decreased the invasiveness of pancreatic CSCs. Further research indicated that metformin downregulated the expression of stemness markers, including Oct4, EpCAM, Nanog, CD44, Notch 1 and Oct4. Additionally, its effect on miRNA was noted: after therapy, let-7a, let-7b, miR-26a, miR-101, miR-200b and miR-200c expression levels were elevated in pancreatic cancer cells [124]. In addition, metformin can induce the so-called metabolic reprograming of CSCs [91]. 

### 8.8. Decitabine and Vorinostat

5-Aza-2′-deoxycytidine (decitabine) and vorinostat, despite belonging to different pharmacological groups, had a rather similar effect on pancreatic cancer. These drugs are able to affect pancreatic CSCs via increasing the expression of miR-34a (Table 1), which is typically downregulated in pancreatic cancer and its stem cells. Re-expression of miR-34a caused by decitabine and vorinostat had a proapoptotic and antiproliferative effect on CSCs, as well as suppressing their self-renewal capabilities. Moreover, vorinostat’s mechanism of action interferes with EMT via lowering the levels of transcription factor ZEB1 that is capable of inducing a transition [114].

**Table 1:** Anti-CSC agents and the relevant therapy targets.

Drug	Therapy Targets	Reference
Salinomycin	Wnt pathway (LARP6), CD133+ cells	[115,116,117]
Gramicidin A	Mitochondrial ultrastructure	[118]
Chloroquine	Hedgehog pathway (Smo)	[119]
Aspirin	NF-κB	[120]
Disulfiram	ALDH, NF-κB	[122]
MT110	EpCAM+ cells	[123]
Metformin	NF-κB, MAPK/mTOR	[91,124]
Decitabine	miR-34a	[114]
Vorinostat	miR-34a, ZEB1	[114]

## 9. Conclusions

The poor five-year survival rate and the rising incidence rate are factors that identify pancreatic cancer as an unmet medical need and, as a result, an important topic of research. Its inherent resistance to conventional therapeutic agents is partially attributable to cancer stem cells: a minor population (0.2–0.8%) of quiescent, self-renewing and pluripotent malignant cells. In pancreatic ductal adenocarcinoma, cancer stem cells were first demonstrated in 2007. Since then, several molecular cascades have been found to be activated in CSCs, including Wnt, Hedgehog, Notch, Hippo and JAK/STAT pathways. CSC are associated with epithelial–mesenchymal transition and, thus, with a metastatic spread of the tumour. Stem cells of pancreatic ductal adenocarcinoma are characterised by a frequent presence of the *K-Ras* mutation, a wide spectrum of epigenetic events and an altered spectrum of microRNAs. Surprisingly, controversial data regarding the metabolic needs (aerobic glycolysis versus oxidative phosphorylation) of stem cells of pancreatic ductal adenocarcinoma have been obtained. 

Current therapeutic strategies are not effective at eliminating the stem cell population. Experimental anti-CSC agents are emerging that target the activated molecular pathways, cellular organoids and/or microRNAs.

## Data Availability

Not applicable.
